# Spatial Organization and Dynamics of Transcription Elongation and Pre-mRNA Processing in Live Cells

**DOI:** 10.4061/2011/626081

**Published:** 2011-11-24

**Authors:** Miguel Sánchez-Álvarez, Noemí Sánchez-Hernández, Carlos Suñé

**Affiliations:** ^1^Dynamical Cell Systems Team, Section of Cellular and Molecular Biology, The Institute of Cancer Research, London SW3 6JB, UK; ^2^Department of Molecular Biology, Instituto de Parasitología y Biomedicina “López Neyra” (IPBLN-CSIC), 18100 Armilla, Spain

## Abstract

During the last 30 years, systematic biochemical and functional studies have significantly expanded our knowledge of the transcriptional molecular components and the pre-mRNA processing machinery of the cell. However, our current understanding of how these functions take place spatiotemporally within the highly compartmentalized eukaryotic nucleus remains limited. Moreover, it is increasingly clear that “the whole is more than the sum of its parts” and that an understanding of the dynamic coregulation of genes is essential for fully characterizing complex biological phenomena and underlying diseases. Recent technological advances in light microscopy in addition to novel cell and molecular biology approaches have led to the development of new tools, which are being used to address these questions and may contribute to achieving an integrated and global understanding of how the genome works at a cellular level. Here, we review major hallmarks and novel insights in RNA polymerase II activity and pre-mRNA processing in the context of nuclear organization, as well as new concepts and challenges arising from our ability to gather extensive dynamic information at the single-cell resolution.

## 1. Introduction

In eukaryotic cells, the regulation, expression, and subsequent processing steps of genomic sequences tend to be localized to defined spaces within the nucleus [1]. In the interphase nucleus, uncondensed chromosomes do not expand randomly but occupy defined volumes termed “chromosome territories,” whose relative positioning has recently been suggested to be determined by, or at least correlated with, differentiation stages and specific contexts [2–4]. This architecture facilitates the intermingling of specific subsets and combinations of genes that need to be coregulated in a given situation [4–7]. Indeed, active genes are most often positioned in the periphery of chromosome territories, while inactive genes remain located within more inaccessible areas of these regions. Although the molecular basis for this dynamic behavior of chromatin is not yet well understood, there are a significant number of studies supporting this concept, thus suggesting a novel layer of complexity in the regulation of gene expression. Genes are not inert entities waiting for the adequate subset of transcription factors to initiate the assembly of a processive RNA polymerase II (RNAPII) complex; instead, the dynamic positioning contributes to their activation state and correlation with other gene units and regulatory elements, such as enhancers and insulators [8–11]. 

Chromosome territories delimitate a region of the nucleus (usually termed the interchromatin space) that is relatively empty of dense chromatin and is hypothesized to be highly interconnected across the nucleus with a higher-order organization [12–15]. The delimited interchromatin volume contains not only most of the transcriptional activity at its boundaries but also contains several nonmembrane-bound dynamic structures—nuclear bodies—highly enriched with specific subsets of nuclear factors [16–18]. These nuclear bodies include Sam68 bodies, PML bodies, paraspeckles, Cajal bodies, and nuclear speckles.

## 2. The Transcription Factory: A Spatial and Functional Unit for RNAPII Transcription

In the early 1990s, two groups reported the use of novel techniques that allowed the visualization of transcriptionally active sites within the nucleus through the incorporation of bromo-UTP in nascent transcripts [19, 20]. In these experiments, nascent transcripts remained immobilized at the site of their chromatin template, and they were studied in great detail using confocal and electron microscopy. Notably, the number of observed active sites appeared to be considerably lower than the estimated number of active molecules of RNAPII [21–23]. These discrete structures colocalize with hyperphosphorylated forms of RNAPII and are resistant to DNA digestion and extraction of soluble fractions [20, 21, 24–26]. These results suggest the existence of an immobile pool of hyperphosphorylated RNAPII within the eukaryotic nucleus. Subsequent fluorescence recovery after photobleaching- (FRAP-) based experiments performed on cells expressing a GFP-tagged construct of RNAPII support this interpretation [27]. Given that the number of observed transcription sites was significantly lower than the number of elongating RNAPII molecules as assessed by *in vitro* run on assays [22, 23], a model was proposed in which several active (mostly elongation-competent) RNAPII units assemble into higher-order structures termed “transcription factories” [21]. According to this model, chromatin loops are tethered to the factories through RNAPII or/and transcription factors for transcription to occur (recently reviewed in (Cook, [28])).

This model is consistent with the looped conformation model that several other independent approaches have suggested exists for an active eukaryotic gene [29–31]. In this model, upon recognition and activation by specific factors, the promoter sequence of the gene unit is tethered to the RNAPII subunit of the factories, and this attachment would be maintained during the transcription of the whole gene sequence, which is “reeled” on the RNAPII. This arrangement provides an additional layer of control and coordination over the different stages of transcription and positions the RNAPII units for subsequent rounds of transcription.

The existence of factories provides us with an elegant conceptual framework to explain the coregulation of functionally related groups of genes in specific contexts [30]. It has been observed that some of these active genes tend to be found in close proximity at a much higher frequency than would be expected by chance [32, 33]. Indeed, these genes tend to share a factory when they are positioned in close proximity, as observed by immunolabeling elongating RNAPII [33]. Although the structural resolution of transcribed genes is still technically limited, nevertheless, this reflects the potential crosstalk that can exist between the transcription factors recruited to each coregulated promoter. Some of the examples consistent with this model are the NF-*κ*B/TNF*α* activation axis [34, 35] and the ER*α* module system [34]. Moreover, genes of different sizes and elongation timing coimmunoprecipitate with the elongating form of the RNAPII in a fashion consistent with the model in which they share the same factory and slide along the “polymerase reading heads” in sequential rounds of transcription, rather than just recruiting mobile polymerase complexes from proximal storage sites and undergoing independent read-throughs [35]. Transcription factories are also consistent with data suggesting that genes with shared features, such as promoter composition and the presence or absence of introns, tend to associate among each other [36]. Finally, transcription factories also provide an explanation for observations that indicate that promoter composition and associated events can influence subsequent stages of transcription elongation [37, 38].

Recent studies have reported on the stability of RNAPII foci upon disrupting transcription [39]. Interestingly, treatment of cells with 5,6-dichloro-1-*β*-d-ribofuranosylbenzimidazole (DRB; a highly specific inhibitor of the positive elongation transcription factor, P-TEFb, and thus an inhibitor of elongating polymerases) does not abolish the association of previously engaged genes with the RNAPII foci, at least for erythroid lineage-specific genes [39]. Observations in agreement with this model include independent genome-wide chromatin immunoprecipitation- (ChIP-) based studies that demonstrate that a significant number of genes is “primed” for transcription. These inactive genes have paused RNAPII complexes at their promoter regions and, upon gene activation, are released from the paused state, allowing elongation to proceed [40].

Initial studies using *in situ* spectroscopy have recently been carried out to determine the composition of the transcription factories [41]. In these studies, the authors demonstrated the existence of clearly defined ribonucleoprotein structures that coincide with sites of active transcription (the perichromatin fibrils), as assessed by BrU pulse incorporation and immunogold labeling. The size and estimated composition of carbon and nitrogen in these structures support the existence of the proposed model of assembled transcription factories, creating a more refined structural model in which the effector subunits of the RNAPII face outwards [41, 42]. 

Another important feature of transcription factories is the enhancement of the physical and functional coupling of transcription and downstream RNA processing steps. This is facilitated by the regulated recruitment of neighboring machinery for cotranscriptional mRNA maturation in an appropriate fashion and timing. This notion would expand the category of these structures to integrated “mRNA factories,” providing an intuitive physical framework for the numerous observed interactions among transcriptional and mRNA processing factors [43–46]. Other essential processes in the regulation of pre-mRNA synthesis, such as chromatin remodeling and histone modification, would similarly benefit from such a design [30].

**Figure 1:**
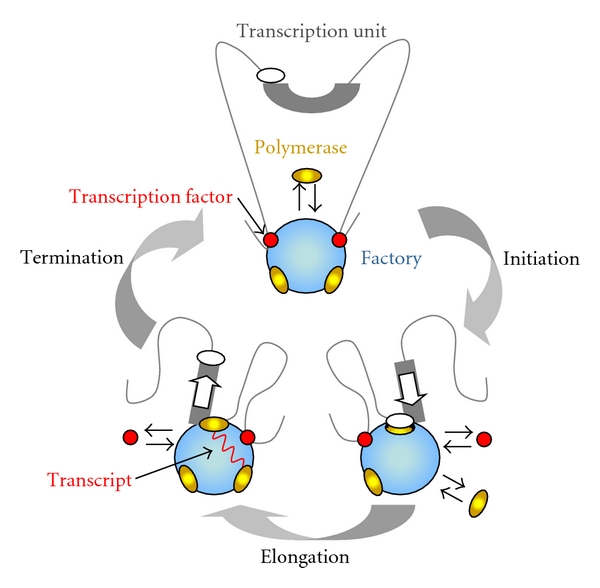
Model of the basic structure and function of RNAPII transcription factories. (a) A “gene-loop” is recruited upon activation to the transcription factory, which contains immobilized subunits of RNAPII. (b) The gene-loop is then “reeled” onto RNAPII for transcriptional elongation. (c) Upon termination, the anchoring of the locus allows for subsequent rounds of transcription. Adapted, with permission, from The Journal of Cell Science [47].

Although this model of transcription factories (Figure 1) explains many observations of gene expression and nuclear organization, there are many intriguing questions that need to be resolved. What are the molecular mechanisms underlying the appropriate targeting of activated gene sequences to a factory, and how are they integrated in a given regulatory context? What is the inner structure of factories in the cell at a resolution beyond the conventional light diffraction limits? How are these structures assembled and organized during the cell cycle according to the requirements of the cell? Does it require the existence of an underlying structural scaffold or “nucleoskeleton?” How are different regulatory hallmarks, such as the phosphorylation cycle proposed for the CTD of the RNAPII during its progression through the transcription of a gene unit, integrated into the context of these structures? Finally, what is the functional relationship between transcription factories and other nuclear compartments related to the biogenesis of the mRNA? 

## 3. Nuclear Speckles and the Regulation of Transcription and Pre-mRNA Processing

Many independent studies in the last two decades have led to a model in which the maturation of nascent transcripts take place simultaneously to their synthesis, that is, cotranscriptionally [48, 49]. This may be specific to a subset of genes or even to specific introns of a gene and is therefore considered not to be strictly required for the completion of pre-mRNA processing itself [50]. However, cotranscriptional processing allows for the functional coupling of the different steps of RNA biogenesis. The bidirectional interdependence among chromatin conformation and posttranslational modifications, in both transcription and different steps of pre-mRNA processing, constitutes an additional layer in gene expression regulation [51–56]. Moreover, it may play a pivotal role in complex processes, such as neuronal differentiation and activity, global integration of RNA processing signatures and DNA damage, and developmental programs [57–60]. 

If pre-mRNA processing is performed largely in a regulated cotranscriptional fashion, the dynamic distribution of pre-mRNA processing factors should be correlated with the organization of transcriptionally active sites in the nucleus. The distribution of pre-mRNA processing factors in the eukaryotic nucleus, as observed using immunofluorescence staining and light microscopy, is not homogeneous and shows a dynamic pattern of localized accumulation in 10–30 irregular domains termed speckles, “SC35 domains” or “splicing factor compartments” (SFCs). At the level of electron microscopy, they correspond to two distinct structures: (i) interchromatin granules clusters (IGCs), composed of particles measuring 20–25 nm in diameter and (ii) perichromatin fibrils, 3–5 nm fibrillar structures localized both at the periphery of IGCs and in other nucleoplasmic regions, which are the sites of nascent pre-mRNAs (for extensive reviews, see [61, 62]) These structures were first identified using immunostaining with specific antibodies against different small nuclear ribonucleoproteins (snRNPs) [63, 64]. This and other observations that show the presence of poly(A) RNA colocalizing with snRNPs and SC35-rich domains [65, 66] further support a link between nuclear speckles and pre-mRNA metabolism. Pioneering mass spectrometry studies [67, 68] and a still-growing list of publications using immunofluorescence or live-cell imaging labeled with tagged constructs corroborate a marked enrichment of these compartments with factors involved in pre-mRNA transcription and processing, especially pre-mRNA splicing. 

**Figure 2:**
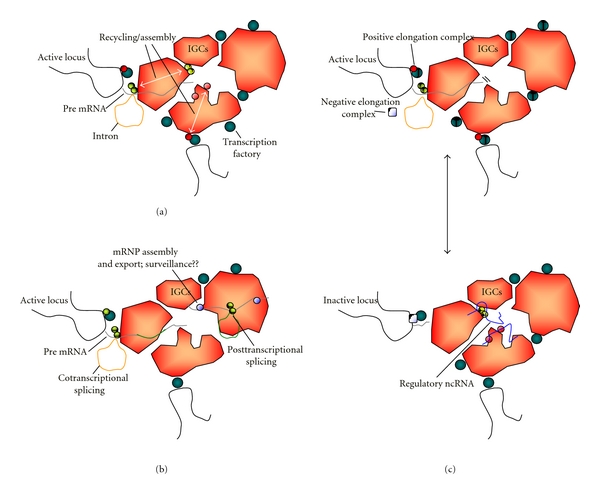
Three potential, nonexclusive models for the role of the interchromatin granule clusters (IGCs) in the regulation of transcription and pre-mRNA processing machinery. IGCs are depicted surrounded by transcriptionally competent sites or “factories” (grey beads). (a) IGCs may be specialized sites for the recycling and assembly of transcription (dark and light red beads) and pre-mRNA processing complexes (dark and light green beads) through regulated posttranslational modification cycles. Dark and light hue code denotes active and inactive pools of factors, respectively. (b) Posttranscriptional processing steps and potential surveillance of mRNA quality may be integrated in these structures, constituting a “checkpoint link” between mRNA transcription and mRNP assembly and export. A given transcript may include both introns that are spliced cotranscriptionally outside of the IGCs (orange lariat) and intron sequences that are processed posttranscriptionally (dark brown stretch and lariat). The later event may be also coupled in the IGCs to surveillance mechanisms, mRNP assembly (blue beads), and export. (c) Specific subsets of nuclear factors, such as ncRNAs (MALAT1, 7SK; see main text; depicted in the lower panel as thin blue threads), can function as active quenchers or sequesters of transcription and pre-mRNA processing factors (red and green beads, resp.), blocking the recruitment of these complexes from the IGCs to nearby active sites of transcription.

Several models, which are not mutually exclusive, have been proposed to explain the role of these nuclear bodies in the regulation of gene expression: (1) they function as storage/assembly/modification compartments that can supply processing factors to the surrounding active transcription sites; (2) they function as sequestering sites for the dynamic control of transcription and processing factors; (3) they serve as functional “hubs” for coregulated genes and their products; (4) they play an active role in posttranscriptional pre-mRNA processing and surveillance and/or in the coupling of early steps of mRNA biogenesis (Figure 2).

 The concept that nuclear speckles are transcriptionally inactive compartments that serve as storage or recycling sites of pre-mRNA metabolism complexes from which these complexes are recruited to nearby sites of active transcription according to demand is a widely held view supported by many experimental results [69–77]. Importantly, recruitment to active sites of transcription requires the integrity of the carboxyl terminal domain of the RNAPII [78], which indicates that transcriptional elongation plays a critical role in the recruitment of pre-mRNA processing factors. 

This proposal is compatible with the view that speckles act as inhibitory sites where specific factors are actively sequestered when their functional repression is required. The essential splicing factor SRSF1 is sequestered into these regions upon the induction of stress through a mechanism dependent on the dynamic interaction of SRPK with stress chaperone complexes, including Hsp70 and Hsp90 [79]. Similarly, the *MALAT1* large noncoding (nc) RNA has been proposed to regulate the phosphorylation-dependent dynamics of splicing factors and their equilibrium between nucleoplasm availability and nuclear speckle sequestration [80]. Linking transcriptional elongation control to this model, the 7SK small ncRNA, which is a scaffold component of transcription elongation CDK9-CycT1 inactive complexes together with HEXIM proteins (see below), has been proposed to function, at least partially, by sequestering these inactive P-TEFb complexes at nuclear speckles [81]. However, there is currently little information known about the actual relevance of these dynamic interactions regarding the response of the cell to specific stimuli or its correlation with changes in transcriptomic profiles. For example, P-TEFb components colocalize at nuclear speckles with these negative regulators and also with the transcription activator adaptor Brd4 [82]. Rigorous quantitative approaches, as opposed to qualitative descriptions, especially those addressing the kinetics of these interactions, such as FRET-based studies, may complement these lines of research. 

Nuclear speckles are most often adjacent to a relatively high density of transcriptionally active regions [21, 25, 46, 71, 83–85], and these active sites mostly represent elongation-competent complexes. Many of these active units correspond to specific, functionally interrelated protein-coding genes, and their juxtaposition to speckles may constitute an important part of their functional program, as has been suggested for genes involved in muscle differentiation [86]. A proposed role for nuclear speckles in these associations is the recruitment of splicing factors at specific active genes in early G1 phase, signaling for the subsequent recruitment of other functionally related gene units later on in the cell cycle [87, 88]. However, this model may be incomplete for explaining the highly dynamic behavior of these structures as observed by live-cell imaging. The inducible recruitment of active genes compared to the dynamics of nuclear speckles has indeed been observed in live cells recently [89]. The authors proposed the following three different, nonmutually exclusive interpretations for this dynamic association such that taking the induced locus as a reference, the speckles could either be (i) assembling *de novo*, (ii) gathering by “coalescence” of smaller speckles, or (iii) recruiting the active gene to their surface. Interestingly, this inducible spatial correlation was dependent on the integrity of the inducible promoter driving the construct, *Hsp70*, which is known to be regulated by the activity of this model gene at the stage of elongation. Again, these observations might indirectly support a functional coordination between transcriptional elongation and the recruitment of pre-mRNA processing machinery. It remains to be fully resolved whether the nuclear speckles have an active role in the higher-order organization and functional coordination of the expression of specific genes or whether they rather arise as a consequence of the spatial concentration of required factors in areas proximal to active, coregulated genes. Importantly, it also remains to be elucidated if there is any posttranscriptional advantage for coregulated genes to converge at the same speckle. It would be interesting to unravel, for example, if subsets of genes that preferentially localize to the nuclear speckle periphery are enriched in genes that are mostly regulated at the level of elongation and whether both their synthesis and processing are enhanced upon appropriate recruitment to these compartments. 

 What is the behavior of the synthesized pre-mRNAs and mRNAs as related to nuclear speckles? A majority of introns are spliced, presumably in a cotranscriptional fashion, outside of the nuclear speckles [90–92]. This peripheral region of nuclear speckles can be therefore considered a potential interphase for cotranscriptional pre-mRNA processing. However, some introns undergo posttranscriptional processing, and their relative accumulation can be observed in these compartments, as in the case of intron 26 of the COL1A1 gene. Mutations that alter the splicing of this intron provoke increased accumulation of the transcript in nuclear speckles [91]. These data raise the possibility that nuclear speckles have a role in posttranscriptional or even postmaturation steps linked to mRNA surveillance and/or nuclear export pathways [90, 93]. Recently, a role for these structures has been proposed in the regulation of posttranscriptional “quality assessment” and the export of Herpesvirus mRNAs [94]. In this case, inducible recruitment of “viral transcription factories” to the peripheral areas of these structures was observed. Bimolecular fluorescence complementation (BiFC) experiments, showing the interaction between the exon-junction complex component Y14 and nuclear export factor 1 (NXF1) and their significant accumulation in nuclear speckles and peripheral areas, further indicate that export-competent spliced mRNAs localize at speckles and that this domain might play an active role in mRNA processing, including maturation and/or transport [95, 96]. This model is also compatible with the apparent subcompartmentalized organization of nuclear speckles [97, 98], which renders a “sponge-like” or “porous” structure that would easily allow for the transit of macromolecular complexes through its interior [99]. Coupling transcription elongation and mRNP assembly with export has been described in yeast and humans, and its potential relationship with the dynamics of mRNP transit through the speckles has been suggested recently [100–104]. Nuclear speckles may represent specialized compartments for the appropriate regulation and coordination of these functions.

Intriguingly, although transcription does not take place within nuclear speckles, a large subset of transcription factors accumulates in these regions, and transcription elongation factors are specifically enriched in these regions. RNAPII is also found to be associated with these compartments [44, 68, 105]. Quantitative laser confocal analysis of ultrathin cryosections has shown that nuclear speckles do not act as major storage sites for inactive complexes, but they instead contain a minor, stable pool of RNAPII molecules phosphorylated at the serine 2 residue of the carboxyl-terminal domain (CTD). Importantly, this subpopulation of RNAPII-2pSer is insensitive to DRB treatment [85, 105]. This fact and the absence of *de novo* synthesis of transcripts at nuclear speckles, as determined by UTP analogue incorporation, suggest that these hyperphosphorylated forms of RNAPII are not engaged in active transcription. An intriguing interpretation might be that these RNAPII molecules serve as a platform for posttranscriptional splicing of transcripts that are trafficking through the speckles. 

## 4. Gene Expression Regulation at the Single-Cell Resolution: Studying the Kinetics of RNA Biogenesis

The study of spatial and dynamic properties is essential for understanding gene expression regulation. Techniques, such as FRAP and fluorescence loss in photobleaching (FLIP), are used to obtain very detailed information about diffusion rates, residency times, or proportion of immobile or stably tethered subpopulations of a given molecule in a delimited volume in the cell at a very high temporal resolution. Fluorescence Resonance Energy Transfer (FRET) and fluorescence lifetime imaging microscopy (FLIM), BiFluorescent complementation (BiFC), and specific applications based on fluorescence correlation spectroscopy (FCS) allow the monitoring and semiquantitation of close (mostly direct) interactions between molecules and the mapping of such interactions in relation to different structures of the cell. The use of these techniques in the study of transcription and pre-mRNA processing has led to interesting new concepts regarding their regulation and nuclear organization [106–112]. 

Not all nuclear factors freely diffuse through the nuclear space. The movement of some of these are compatible with a model in which factors “scan” unspecific genomic sequences or/and bind components of the RNA machinery through a weak and transient binding until they engage in a favorable, specific assembly on their target sites [113]. Nuclear functions and organization likely arise not from the static state of their components but from an extremely dynamic equilibrium between multiple functional interactions [10, 114]. Rino and coworkers [115] found that nuclear speckles acquired a rounder and more quiescent morphology, as expected, upon transcription elongation inhibition using the P-TEFb inhibitor DRB. However, when studying the interchange rate of molecules bound to nuclear speckles within the nucleoplasm pool using FLIP, they found that the fluorescence was lost at a higher speed than it was in cells that had not been treated with DRB. Similar studies have been conducted for the transcription elongation complex P-TEFb in the context of Tat-mediated transactivation of the HIV-1 [53, 116]. Interestingly, these studies suggest that a potential mechanism by which Tat might contribute to P-TEFb-mediated transactivation is through the stabilization of CDK9 binding to the transcription site, increasing its residency time by almost tenfold. These observations suggest that cellular factors are constantly engaged in dynamic and highly transient interactions even within some apparently static structures. 

Specific interactions among different spliceosomal components have also been studied in the context of nuclear organization and live-cell behavior using FRET and FLIM techniques. The dynamic model mentioned above is also compatible with the presence of preassembled subcomplexes, such as the spliceosomal components, which can be dynamically recruited to form even higher-order functional complexes [114]. Of note, these subcomplexes exist in even the absence of ongoing transcription and in nuclear compartments in which they do not actively function [75, 115, 117]. 

Recent adaptations of these techniques have been used to study fluorescent proteins that interact with high affinity and specificity to DNA and RNA sequences, such as the LacZ bacterial repressor and the MS2 and PP7 nucleocapsid coating proteins, respectively. The use of engineered constructs containing several tandem repeats of these target elements allows for the efficient recruitment of fluorescent molecules to the desired chromatin template or nascent transcript, amplification of the signal, and reliable detection by conventional light microscopy in living cells. FRAP measurements can then be used to estimate the rates of transcript elongation and release because these stages of mRNA biogenesis correlate with distinct kinetic steps, which can be mathematically dissected with appropriate modeling using the gathered data. Additionally, by combining this transcript-tagging system with the tagging of RNAPII with a distinct fluorophore, the dynamics of promoter binding and transcription initiation can also be inferred using the aforementioned data of transcript synthesis kinetics [118]. Interestingly, transcription appears to be a rather inefficient process, as only approximately 1% of recruited RNAPII molecules are engaged in processive transcription; however, these figures may vary considerably depending on the genetic model studied [119, 120]. These types of studies have led to an estimation of the rate of elongation for the human RNAPII of *∼*4.3 kb/min, although these figures can also vary widely depending on the experimental setting used [118, 121]. For example, a study based on the HIV-1 gene yielded unexpectedly long pausing times for RNAPII units located at the proximal promoter and 3′ terminal regions with an estimated elongation rate of *∼*1.7 kb/min [53, 119]. An important, unresolved issue is the unification of the mathematical models used to infer the kinetic properties of transcription elongation because considering or excluding certain phenomena in the reference model can drastically affect the entire interpretation [121].

These tools have been used not only for characterizing transcription dynamics, but also for facilitating the study of the *in vivo* functional coupling between transcription elongation and pre-mRNA processing, allowing us to obtain novel insights into the basis of pre-mRNA processing in the living cell. For example, it has been determined that early-spliceosomal components are actively recruited to transcribing genes lacking intronic sequences [122]. These observations are in accordance with previous biochemical and functional studies that describe a stable interaction between the initiating RNAPII complexes and the U1 snRNP [123]. In fact, stepwise cotranscriptional recruitment of the spliceosome has been reported recently [124]. Importantly, global splicing inhibition did not prevent recruitment of spliceosomal components to the active transcription site, further supporting that nuclear organization and coordination of pre-mRNA metabolism are significantly determined by transcription.

Finally, innovative microscopy and spectroscopy tools, coupled with powerful statistical analysis and modeling, have led to the first studies in estimating the dynamics of transcription at the single-molecule resolution [121, 125]. These novel approaches will allow us to gain further insights regarding single-cell behavior, and the aspects of noise, robustness, and cell-to-cell variability in pre-mRNA formation and processing, which may be important to globally understand the regulation of gene expression.

## 5. Concluding Remarks

The quantitative study of the spatial and dynamic aspects of transcription and pre-mRNA processing is revealing itself as an essential complement to well-established, classical biochemistry-based approaches to fully understand how the regulation of gene expression is exerted in the cell. As stated in this review, many recent insights that help to explain long-standing questions regarding mRNA biogenesis could not have been achieved otherwise. However, these studies also give rise to important new questions. What is the functional relevance of spatial organization and regulation of dynamics in the different stages of mRNA biogenesis for the cell in a given context? Can we obtain a genome-wide picture of these parameters for all protein-coding genes in a systematic manner? How is cell-to-cell variability regulated within a cellular population to be advantageous for the cellular population as a whole? Is the dynamic regulation of the spatial distribution of the involved factors an essential component for the fine tuning of functional coupling of transcription elongation and pre-mRNA processing? It is expected that there will be a remarkable increase in the usage and optimization of these approaches, combined with more conventional biochemical and functional approaches, in the study of all aspects of mRNA formation and function.
